# Evaluation of Differentially Expressed Genes in Leaves vs. Roots Subjected to Drought Stress in Flax (*Linum usitatissimum* L.)

**DOI:** 10.3390/ijms241512019

**Published:** 2023-07-27

**Authors:** Ningning Wang, Fan Qi, Fu Wang, Yujie Lin, Chunxiao Xiaoyang, Zhanwu Peng, Bi Zhang, Xin Qi, Michael K. Deyholos, Jian Zhang

**Affiliations:** 1 Faculty of Agronomy, Jilin Agricultural University, Changchun 130000, China; ningningw@jlau.edu.cn (N.W.); fan711998@163.com (F.Q.); 20210047@mails.jlau.edu.cn (F.W.); linyujie97@163.com (Y.L.); xycxttkx@163.com (C.X.); 13919684204@163.com (B.Z.); 2 Information Center, Jilin Agricultural University, Changchun 130000, China; pengzhanwu@jlau.edu.cn; 3 Department of Biology, University of British Columbia Okanagan, Kelowna, BC V1V 1V7, Canada; michael.deyholos@ubc.ca

**Keywords:** oil flax, fiber flax, drought stress, gene expression, photosynthesis, lignin

## Abstract

Drought stress is a common environmental challenge that plants face, severely constraining plant growth and reducing crop yield and quality. Several studies have highlighted distinct responses between monocotyledonous and dicotyledonous plants. However, the mechanisms underlying flax tolerance to abiotic stress, such as drought, remain unclear. In this study, we investigated the morphological, physiological, and biochemical characteristics and the genome-wide gene expression of oil flax and fiber flax in response to drought stress. The results revealed that drought stress caused significant wilting of flax leaves. Within the first 24 h of stress, various physiological and biochemical characteristics exhibited rapid responses. These included fresh weight, relative water content (RWC), proline, soluble protein, soluble sugar, superoxide dismutase (SOD), peroxidase (POD), and catalase (CAT) in the leaves or roots of flax. Additionally, drought stress led to a significant rise in lignin content in fiber flax. In addition, the transcriptome analysis demonstrated genome-wide variations in gene expression induced by drought stress. Specifically, genes associated with photosynthesis, proline biosynthesis, and phytohormone metabolism exhibited significant differences in expression levels under stress conditions in flax. These findings highlight the rapid response of flax to drought stress within a short-term period. Our experiment also revealed that, although there were variations in the levels of small compound content or gene expression between Longya10 and Fany under drought stress, most stress-resistance responses were similar. Furthermore, the results provide additional evidence supporting the existence of mechanisms underlying the response to drought stress in plants.

## 1. Introduction

Flax, an ancient crop cultivated worldwide, can be categorized into three main types: fiber flax, oil flax, and oil-fiber flax [1]. A typical example of oil flax is Longya10, originally from China and widely used as an oilseed material in various sectors such as food, health products, and cosmetics [2,3]. Another notable variety, Fany, originates from Europe and is widely used in the textile and manufacturing industries [1,4]. Oilseed-type flax plants typically have a shorter stature, more branches, and higher seed production, while fiber flax types are generally taller, have fewer branches, and are specifically selected for fiber production [5]. To ensure optimal growth, flax thrives in environments with temperatures ranging from 20 to 25 °C and humidity levels between 60 and 80% [1]. During the early stages of growth, warm and dry climates promote branching and seed setting. Once the vegetative growth phase is completed, dry weather facilitates seed maturation. Sufficient water is essential for flax’s growth, as drought conditions can stunt plant growth and induce lignification [1,6].

Global warming has resulted in widespread land aridification, significantly impacting crop growth [7]. This phenomenon has disrupted plant development and reduced crop yields due to dehydration [8]. Drought stress poses a severe threat to plants, causing extensive dehydration [8]. Consequently, it is crucial to investigate the molecular mechanisms underlying drought stress in order to sustain agricultural productivity amidst changing climatic conditions [9]. However, previous studies that utilized transcriptional or proteomic profiling have shown that short-term plant responses to stress factors can play a crucial role in regulating gene expression and physiological responses [10,11]. For instance, during hormone treatment or salt stress, transcriptional or proteomic changes were observed [10,11]. It was also found that the transcriptional changes caused by short ABA treatment were much stronger than those caused by nine days of treatment, and promoted the expression of genes encoding ethylene and JA signaling components [10]. Given the growth characteristics of flax, it is imperative to study the alterations that occur in flax plants when subjected to short-term drought stress.

Research has indicated that drought stress induces variations in plant morphology, physiological characteristics, gene expression, and protein conformation [12,13,14]. These changes have a significant impact on photosynthesis efficiency, accelerating plant senescence and ultimately leading to plant death [15]. The reduced water concentration surrounding plant cells triggers osmotic pressure, causing water molecules to move out of the cells. To maintain stable internal osmotic potential, cells synthesize and accumulate various small organic compounds, such as proline, soluble proteins, and soluble sugars. These compounds contribute to maintaining the accuracy of protein folding and the stability of macromolecular structures [16].

Proline accumulation is a notable metabolic response of plants to drought stress [17]. Studies have shown that overexpressing genes from the P5CS or the P5CR family, which are involved in proline biosynthesis, can enhance plants’ drought tolerance [18]. Simultaneously, osmotic stress triggers the accumulation of reactive oxygen species (ROS), causing severe damage to cellular structures, known as oxidative stress [19]. To counteract this, plants increase the production of antioxidant enzymes and antioxidants. Examples of these include SOD, CAT, and POD [16]. These enzymes play a crucial role in scavenging ROS and protecting cellular structures, including the membrane, by reducing the levels of malondialdehyde (MDA) produced from lipid peroxidation [16].

Drought stress profoundly impacts the structure of plant cell walls [20,21]. The osmotic pressure induced by drought causes water to permeate from inside to outside the cells until a balance is achieved [22]. As a result, the stem becomes thinner and weaker, leaves turn soft and yellow, the entire plant wilts significantly, and plasmolysis occurs in plant cells [23]. To prevent mechanical damage caused by cell wall collapse due to dehydration and osmotic stress reduction, the cell wall undergoes thickening and lignification to adapt to the changing environment [24]. The cell wall constitutes a principal structural component of vascular plants, comprising lignin, cellulose, hemicellulose, and pectin. Its role in furnishing structural rigidity facilitates upright growth and enhances resilience against excessive transpiration due to abiotic stress and biotic stress arising from pathogenic bacterial infections. Consequently, environmental variations have the potential to significantly perturb the biosynthesis of lignin, cellulose, hemicellulose, and pectin [25,26].

The monomers involved in lignin biosynthesis are coniferyl alcohol, sinapyl alcohol, and coumaryl alcohol. Through polymerization, these monomers produce guaiacyl (G unit), syringyl (S unit), and hydroxyphenyl (H unit), respectively [25]. The lignin biosynthesis pathway originates from phenylalanine and is extensively studied in plants [25]. Various genes and enzymes participate in each step of this pathway, including phenylalanine ammonia-lyase (PAL), cinnamate 4-hydroxylase (C4H), 4-coumarate CoA ligase (4CL), shikimate/quinate hydroxycinnamoyl transferase (HCT), coumarate 3-hydroxylase (C3H), caffeoyl CoA O-methyltransferase (CCoAOMT), ferulate 5-hydroxylase (F5H), caffeic acid O-methyltransferase (COMT), cinnamoyl CoA reductase (CCR), and cinnamyl alcohol dehydrogenase (CAD). Finally, through the catalysis of peroxidases and laccases, various lignin monomers polymerize into lignin [25].

Cellulose, the most abundant component of the cell wall, accounts for approximately 30–40% of its dry weight. It forms a framework of cellulose microfibrils that are cross-linked by xyloglucans and pectin polysaccharides [26,27]. Cellulose is a 1,4-β-D-glucose polymer, and the substrate for cellulose biosynthesis is UDP-glucose, and the biosynthesis process requires plasma membrane-localized cellulose synthase complexes (CSCs) consisting of cellulose synthase A (CESA) subunits.

Hemicellulose is a heteropolysaccharide composed of different types of monosaccharides, primarily including Xyloglucan, Xylan, and Mannans [28]. The large glycosyltransferase 2 (GT2) superfamily includes cellulose synthase-like enzymes (CSLs), which consist of nine subfamilies (CSLA–H and CSLJ). CSLC family members are involved in xyloglucan biosynthesis, the GT43 glycosyltransferases family (IRXs) are required for xylan backbone biosynthesis, and the CSLA family members are implicated in mannan biosynthesis [29,30].

Phytohormones play a critical role in regulating plant growth and development. In the face of dehydration and osmotic stress, the levels of endogenous hormones rapidly respond to environmental variations [31]. One prominent hormone involved in this response is abscisic acid (ABA). ABA regulates stomatal opening and closure [32,33], thus impacting plant transpiration, and it also mediates the content of ROS by regulating calcium ion (Ca^2+^) channels in guard cells [34,35]. In addition to ABA, other phytohormones such as ethylene (ET), auxin (indole-acetic acid, IAA), cytokinin (CK), salicylic acid (SA), jasmonic acid (JA), gibberellic acid (GA), and benzylaminopurine (BA) also interact and cross-talk with ABA in response to drought stress [36,37]. The genes involved in phytohormone metabolism, biosynthesis, degradation, and signal transduction, as well as those induced, regulated, and activated in response to drought stress, have been extensively studied [36,38]. These genes play crucial roles in the plant’s ability to respond and adapt to drought stress [36,38]. Therefore, investigating the variation in gene expression related to phytohormones following drought stress is of utmost importance.

In this study, our aim is to investigate the immediate response of oil and fiber flax to short-term drought stress. We analyze the changes in morphology, physiological characteristics, and gene expression patterns under drought conditions. The study has three primary objectives: (1) We aim to identify and document the specific changes in plant morphology that occur as a result of drought stress. (2) We will focus on understanding the impact of small organic compounds in response to drought stress. (3) We will examine changes in gene expression patterns to comprehend the molecular processes underlying the response to drought stress in both oil flax and fiber flax. Through these analyses, we aim to shed light on the short-term response of oil and fiber flax to drought stress, providing valuable insights into morphological, physiological, and genetic aspects of their adaptation mechanisms.

## 2. Results

### 2.1. The Morphological Characters and Small Organic Compounds Contents Were Altered following Drought Stress in Flax

**Figure 1:**
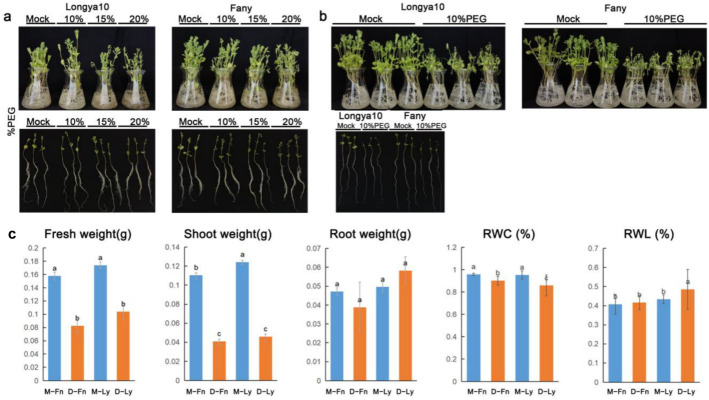
(**a**) Drought stress was induced using different concentrations of PEG solution (0%, 10%, 15%, and 20%) for a duration of 24 h in Fany and Longya10 flax plants. (**b**) Morphological changes in Fany and Longya10 flax plants were observed under 10% PEG solution for 24 h. A comparison was made between the mock condition (control) and the stress condition. (**c**) The following parameters were evaluated in Fany and Longya10 flax plants: shoot weight, fresh weight, root weight, RWC, and rate of water loss (RWL). The abbreviations M-Fn and M-Ly represent flax growth in the mock condition derived from Fany and Longya10 flax, respectively. D-Fn and D-Ly represent flax growth in the stress condition derived from Fany and Longya10 flax. Statistical significance between the two columns is indicated by the letters ‘a’, ‘b’ or ‘c’, denoting a significant difference at *p* < 0.05.

We used the variates of Longya10 and Fany, which were kindly provided by XinJiang University, as the subjects of our investigation. Different studies have shown that induced drought stress through polyethylene glycol (PEG) significantly reduces the rate and percentage of seed germination in plants [39,40]. In line with these prior reports, we simulated drought stress conditions by employing the PEG protocol. The flax plants were cultivated for 18 days in an artificial illumination incubator with temperature conditions set at 26 °C during the day and 18 °C during the night. The plants were divided into three groups, each subjected to a different PEG concentration: 10%, 15%, and 20%. These treatments were administered for a duration of 24 h. The findings of our study revealed that the application of the 10% PEG treatment led to the induction of curling and dehydration in the plants, with the seedlings exhibiting a yellowish hue and drying out rapidly following short-term exposure to stress (Figure 1a). The phenotypic effects were even more conspicuous in the Longya10 and Fany varieties subjected to 15% and 20% PEG treatments (Figure 1a). Consequently, the 10% PEG treatment was deemed suitable for inducing drought stress in flax (Figure 1b). Following 24 h of drought stress, the fresh weight of the seedling flax exhibited a significant decrease, particularly in the shoot organ rather than the root organ (Figure 1c). We observed that the RWC of wilted seedlings was approximately 90% in Fany and 85% in Longya10 after the 24-h drought stress (Figure 1c). Additionally, the rate of water loss was significantly higher in Longya10 after the drought stress (Figure 1c).

### 2.2. The Physiological Characteristics Variation following Drought Stress in Flax

**Figure 2:**
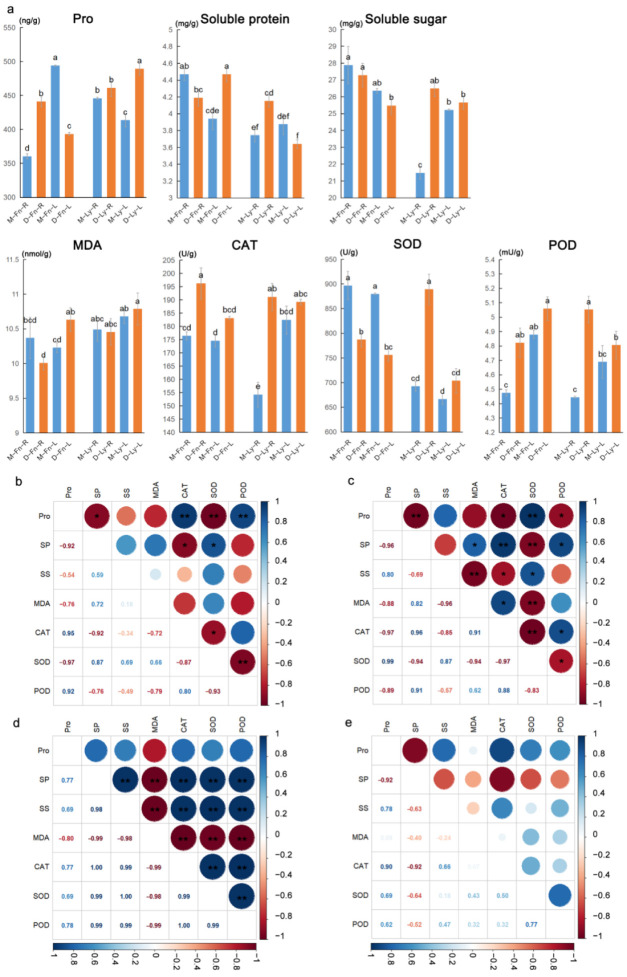
(**a**) The levels of proline, soluble protein, soluble sugar, MDA, CAT, SOD, and POD were compared between Fany and Longya10 flax plants under drought stress conditions. Statistical significance between the two columns is indicated by different letters, representing a significant difference at *p* < 0.05. The abbreviations M-Fn and M-Ly represent flax growth in the mock condition derived from Fany and Longya10 flax, respectively. D-Fn and D-Ly represent flax growth in the stress condition derived from Fany and Longya10 flax. -R represents the root organ of flax, and -L represents the leaves organ of flax. (**b**) Correlation analysis was conducted in the roots of Fany flax plants. (**c**) Correlation analysis was performed in the leaves of Fany flax plants. (**d**) Correlation analysis was carried out in the roots of Longya10 flax plants. (**e**) Correlation analysis was conducted in the leaves of Longya10 flax plants. Asterisks (*) are used to denote statistical significance at *p* < 0.05, while double asterisks (**) indicate significance at *p* < 0.01.

Small organic compounds were measured in the leaves and roots of flax following drought stress. These compounds exhibited a rapid response after stress, but their changes varied across different factors, such as Longya10/Fany, leaves/roots, and stress/control, without a specific pattern observed (Figure 2). All samples were marked with “M-Fn-R” (Mock-Fany-Root), “M-Fn-L” (Mock-Fany-Leaf), “D-Fn-R” (Drought-Fany-Root), “D-Fn-L” (Drought-Fany-Leaf), “M-Ly-R” (Mock-Longya10-Root), “M-Ly-L” (Mock-Longya10-Leaf), “D-Ly-R” (Drought-Longya10-Root), and “D-Ly-L” (Drought-Longya10-Leaf), respectively. Among these, the most significant variations were observed in the roots of Longya10. In comparison to the control group, the drought stress treatment resulted in a significant increase in the levels of solute protein, solute sugar, CAT, and the activities of SOD and POD. Additionally, the content of MDA showed a significant elevation in the leaves of the Fany variety after exposure to drought stress. In the roots of Fany, the content of proline, CAT, and POD activity exhibited a significant increase following drought stress. Notably, the impact of drought stress on the leaves was comparatively less pronounced when compared to its effects on the roots (Figure 2a).

The results of the correlation analysis between proline content and other physiological parameters are presented in Figure 2, along with the corresponding significance tests. In the roots of Fany, proline content exhibited a positive correlation with CAT activity and POD activity (*p* < 0.01), respectively. Additionally, soluble protein showed a positive correlation with SOD activity (*p* < 0.05) (Figure 2b). In the leaves of Fany, proline content showed a positive correlation with SOD activity (*p* < 0.01). Moreover, soluble protein showed a positive correlation with CAT activity (*p* < 0.01) and POD activity (*p* < 0.05). MDA content was positively correlated with CAT activity (*p* < 0.05). Furthermore, POD activity exhibited a positive correlation with CAT activity (*p* < 0.05) (Figure 2c). In the roots of Longya10, the soluble protein content exhibited positive correlations with soluble sugar (*p* < 0.01), CAT activity (*p* < 0.01), SOD activity (*p* < 0.01), and POD activity (*p* < 0.01), respectively. CAT activity was positively correlated with SOD activity (*p* < 0.01) and POD activity (*p* < 0.01) (as shown in Figure 2d). In the leaves of Longya10, proline content showed a positive correlation with CAT activity (Figure 2e).

### 2.3. The Lignin and Cellulose Contents Were Altered following Drought Stress in Flax

**Figure 3:**
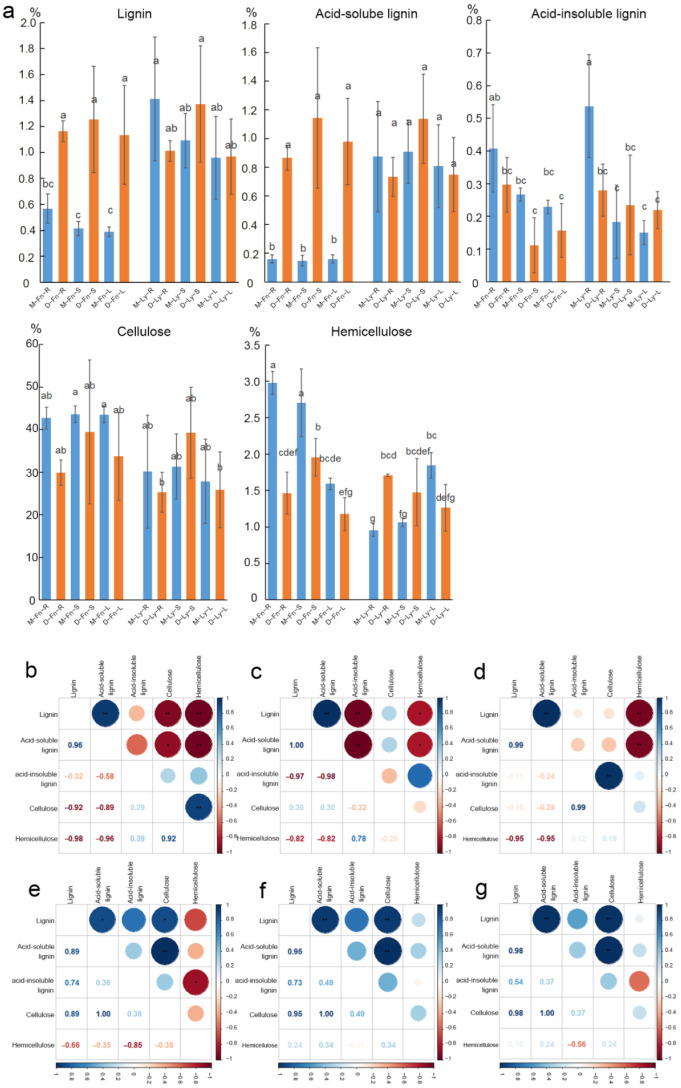
(**a**) The levels of lignin, cellulose, and hemicellulose were compared between Fany and Longya10 flax plants under drought stress conditions. Statistical significance between the two columns is indicated by different letters, representing a significant difference at *p* < 0.05. The abbreviations M-Fn and M-Ly represent flax growth in the mock condition derived from Fany and Longya10 flax, respectively. D-Fn and D-Ly represent flax growth in the stress condition derived from Fany and Longya10 flax. -R represents the root organ of flax, -S represents the stem, and -L represents the leaves organ of flax, respectively. (**b**) Correlation analysis was conducted in the roots of Fany flax plants. (**c**) Correlation analysis was performed in the stems of Fany flax plants. (**d**) Correlation analysis was carried out in the leaves of Fany flax plants. (**e**) Correlation analysis was conducted in the roots of Longya10 flax plants. (**f**) Correlation analysis was performed in the stems of Longya10 flax plants. (**g**) Correlation analysis was carried out in the leaves of Longya10 flax plants. Asterisks (*) are used to denote statistical significance at *p* < 0.05, while double asterisks (**) indicate significance at *p* < 0.01.

In order to examine the changes in lignin and cellulose levels following short-term drought stress in flax, we measured them in different organs, including the root, stem, and leaf of the Longya10 and Fany varieties (Figure 3). Among these, the most pronounced variation was observed in Fany, where the content of lignin increased, and hemicellulose decreased after drought stress. Interestingly, the primary alteration in lignin content was an increase in acid-soluble lignin following stress. On the other hand, the changes in lignin and cellulose content were relatively less significant in Longya10 after stress. Notably, we observed that the content of lignin was higher, the content of hemicellulose was lower, and the content of cellulose was similar in Fany compared to the control group. This difference was particularly notable when compared to the control group of Longya10. These findings suggest that Fany exhibits a more distinct response in terms of lignin and hemicellulose content following drought stress, while the response in Longya10 is relatively less pronounced.

The results of the correlation analysis between lignin content and other physiological characteristics and the results of significance tests are shown in Figure 3. In Fany, the lignin content exhibited a positive correlation with acid-soluble lignin content in the roots (*p* < 0.01), stems (*p* < 0.01), and leaves (*p* < 0.01) (Figure 3b–d). Furthermore, the cellulose content showed a positive correlation with hemicellulose content in the roots (*p* < 0.01) of Fany (Figure 3b). Similarly, in Longya10, the lignin content demonstrated a positive correlation with acid-soluble lignin content in the roots (*p* < 0.05), stems (*p* < 0.01), and leaves (*p* < 0.01) (Figure 3e–g). Moreover, the lignin content exhibited a positive correlation with cellulose content in the roots (*p* < 0.05), stems (*p* < 0.01), and leaves (*p* < 0.05) of Longya10 (Figure 3e–g).

### 2.4. Genome-Wide Gene Expression Was Altered following Drought Stress in Flax

**Figure 4:**
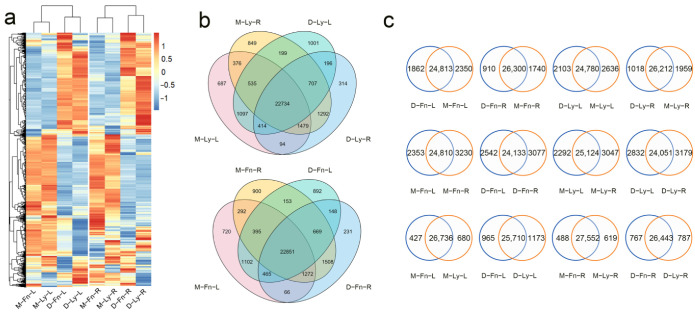
Genome-wide gene expression analysis was conducted on the roots and shoots of Fany and Longya10 flax plants under both mock (control) and drought stress conditions. (**a**) A heatmap was generated to illustrate the gene expression levels in the leaves and roots. The abbreviations M-Fn and M-Ly represent flax growth in the mock condition derived from Fany and Longya10 flax, respectively. D-Fn and D-Ly represent flax growth in the stress condition derived from Fany and Longya10 flax. -R represents the root organ of flax, and -L represents the leaves organ of flax. (**b**) A Venn diagram was created to depict the common and unique gene expression patterns observed in the leaves and roots of Fany and Longya10 flax plants. (**c**) Further details regarding the gene expression patterns between the leaves and roots are provided, with the number of genes marked on each column.

In this study, we conducted RNA-seq profiling to investigate the genome-wide gene expression changes in the roots and leaves of flax following 24 h of drought stress. We obtained approximately 5 gigabases (Gb) of clean data for each sample. To align the clean reads to the reference genome, we used BWA and STAR, as previously reported [41]. The gene expression levels resulting from our analysis are listed in Figure 4a. The results revealed that in the leaves, 27,163, 27,416, 26,675, and 26,883 genes were expressed in the M-Fn-L, M-Ly-L, D-Fn-L, and D-Ly-L samples, respectively. Additionally, in the roots, 28,040, 28,171, 27,210, and 27,230 genes were expressed in the M-Fn-R, M-Ly-R, D-Fn-R, and D-Ly-R samples, respectively. Venn Diagram analysis was performed to identify the overlap and unique expression of genes across the different samples (Figure 4b). For instance, when comparing “D-Fn-L” with “M-Fn-L,” we found that 24,813 genes were expressed in both samples, while 1862 genes were uniquely expressed in “D-Fn-L,” and 2350 genes were expressed exclusively in “M-Fn-L” (Figure 4c). By analyzing the number of overlapping or uniquely expressed genes in each group using the Venn diagram (Figure 4c), we observed that drought stress, tissue/organ specificity, and genotypic specificity induced genome-wide variations in gene expression.

The results of the differentially expressed genes (DEGs) analysis are summarized in Figure S1. The findings are as follows: Comparing the stressed samples with their respective controls, in the leaves of Fany, drought stress induced the upregulation of 5907 genes and the downregulation of 4840 genes. In the leaves of Longya10, there were 7044 upregulated genes and 5566 downregulated genes. In the roots of Fany, 2570 genes were upregulated, and 1477 genes were downregulated. In the roots of Longya10, 3153 genes were upregulated, and 1850 genes were downregulated (Figure S1a). When comparing the leaves with the roots in Fany, under mock conditions, there were 5622 upregulated genes and 5161 downregulated genes. Under stress conditions, there were 5951 upregulated genes and 5919 downregulated genes. Similarly, in Longya10, under mock conditions, there were 5174 upregulated genes and 5157 downregulated genes. Under stress conditions, there were 6875 upregulated genes and 6820 downregulated genes (Figure S1b). Comparing the genotypes Fany and Longya10, under mock conditions, there were 54 upregulated genes in the leaves of flax. Under stress conditions, there were 29 upregulated genes and 25 downregulated genes in the leaves. In the roots, under mock conditions, there were three upregulated genes. Under stress conditions, there were four upregulated genes and six downregulated genes (Figure S1c).

We used Venn Diagram to explore further the overlap-/unique- DEGs in every two groups (Figure S1d). The number of 9239 genes were overlap-expressed in both “D-Fn-L vs. M-Fn-L” and “D-Ly-L vs. M-Ly-L,” 1508 genes unique-expressed in “D-Fn-L vs. M-Fn-L,” and 3371 genes uniquely expressed in “D-Ly-L vs. M-Ly-L”, which manifested that differentially expressed genes in Fany and Longya10 during short term drought stress response.

### 2.5. Gene Ontology (GO) and d Kyoto Encyclopedia of Genes and Genomes (KEGG) Enrichment Analysis following Drought Stress in Flax

**Figure 5:**
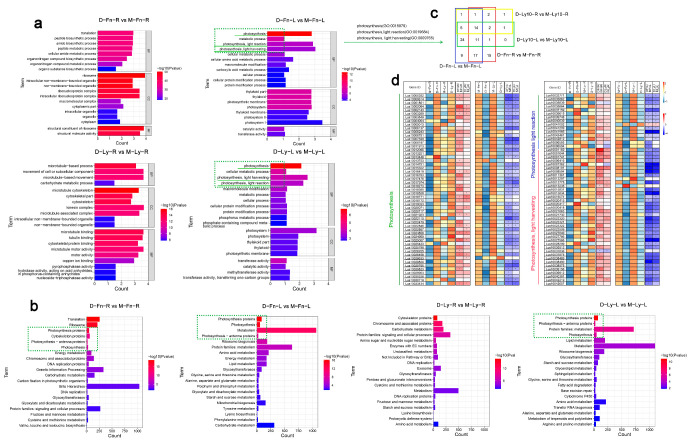
(**a**) Gene ontology (GO) classification and enrichment analysis of the DEGs in Fany and Longya10 flax following drought stress. (**b**) Kyoto Encyclopedia of Genes and Genomes (KEGG) pathways of the significantly enriched DEGs in Fany and Longya10 flax following drought stress. (**c**) The common and unique DEGs between the roots and shoots of Fany and Longya10 flax plants under drought stress conditions were identified. (**d**) A heatmap was generated to visualize the expression levels (in log2 transformed read counts per million) of genes related to photosynthesis in Fany and Longya10 flax plants following drought stress. The abbreviations M-Fn and M-Ly represent flax growth in the mock condition derived from Fany and Longya10 flax, respectively. D-Fn and D-Ly represent flax growth in the stress condition derived from Fany and Longya10 flax. -R represents the root organ of flax, and -L represents the leaves organ of flax. The yellow color indicates the expression levels of the genes, while the white color represents the fold change (log2 value) of the DEGs. Statistical significance is denoted by asterisks (*) with *p* < 0.05.

We analyzed the GO enrichment of DEGs following drought stress in the roots and leaves of flax, respectively (Figure 5a, Table S2). For “D-Fn-L vs. M-Fn-L,” the top enriched GO terms for BP category were photosynthesis-light harvesting (GO:0009765), photosynthesis-light reaction (GO:0019684), and photosynthesis (GO:0015979); those for CC were photosystem I (GO:0009522), photosystem (GO:0009521), and thylakoid (GO:0009579), and those for MF were catalytic activity (GO:0003824), transferase activity (GO:0016740). For “D-Fn-R vs. M-Fn-R,” the top enriched GO terms for BP were translation (GO:0006412), peptide biosynthetic process (GO:0043043), and amide biosynthetic process (GO:0043604); those for CC were ribosome (GO:0005840), ribonucleoprotein complex (GO:1990904), and intracellular ribonucleoprotein complex (GO:0030529), and those for MF were a structural constituent of ribosome (GO:0003735), and structural molecule activity (GO:0005198). For “D-Ly-L vs. M-Ly-L,” the top enriched GO terms for BP were photosynthesis-light harvesting (GO:0009765), photosynthesis-light reaction (GO:0019684), and photosynthesis (GO:0015979), those for CC were photosystem I (GO:0009522), and those for MF were methyltransferase activity (GO:0008168) and transferring one-carbon groups (GO:0016741). For “D-Ly-R vs. M-Ly-R,” the top enriched GO terms for BP were the movement of a cell or subcellular component (GO:0006928), and microtubule-based movement (GO:0007018), those for CC were kinesin complex (GO:0005871), microtubule-associated complex (GO:0005875), and microtubule cytoskeleton (GO:0015630), and those for MF were microtubule motor activity (GO:0003777), microtubule binding (GO:0008017), and tubulin binding (GO:0015631).

To further understand the function of DEGs, we performed a KEGG enrichment analysis (Figure 5b, Table S3). In the comparison between “D-Fn-L vs. M-Fn-L,” significant enrichment was observed in the metabolism and carbohydrate metabolism pathways. In the comparison between “D-Fn-R vs. M-Fn-R,” considerable enhancement was found in the brite hierarchies and genetic information processing. In the comparison between “D-Ly-L vs. M-Ly-L,” there was significant enrichment in the metabolism, amino acid, and lipid metabolism pathways. Finally, in the comparison between “D-Ly-R vs. M-Ly-R,” a considerable increase was observed in metabolism and chromosome-associated proteins.

Compared to the GO and KEGG analyses, the photosynthesis-related genes and protein were enriched following drought stress in flax. We found that genes and proteins related to photosynthesis were enriched following drought stress. Specifically, the processes of photosynthesis (GO:0015979), photosynthesis-light reaction (GO:0019684), and photosynthesis-light harvesting (GO:0009765) showed increased activity in both the “D-Fn-L vs. M-Fn-L” and “D-Ly-L vs. M-Ly-L” comparisons (marked with green dashed box in Figure 5a,b). To further investigate the genes involved in these photosynthesis-related processes, we selected the genes annotated with GO:0015979, GO:0019684, and GO:0009765 and compared them with the DEGs identified in our study. The Venn analysis revealed that 106 genes exhibited altered expression after drought stress in flax, and some of these DEGs were found to be shared between the roots and leaves of flax (Figure 5c). Interestingly, we observed that genes associated with photosynthesis showed higher expression levels in the leaves of flax under normal conditions (Figure 5d, Table S4). However, these genes were significantly downregulated in response to drought stress in the leaves. Conversely, compared to the leaves, the gene expression levels were initially lower in the roots of flax. However, some of these genes were significantly upregulated following drought stress (Figure 5d).

### 2.6. The Expression of Lignin, Cellulose, and Hemicellulose Biosynthesis-Related Genes Was Found to Be Altered in Response to Drought Stress in Flax

**Figure 6:**
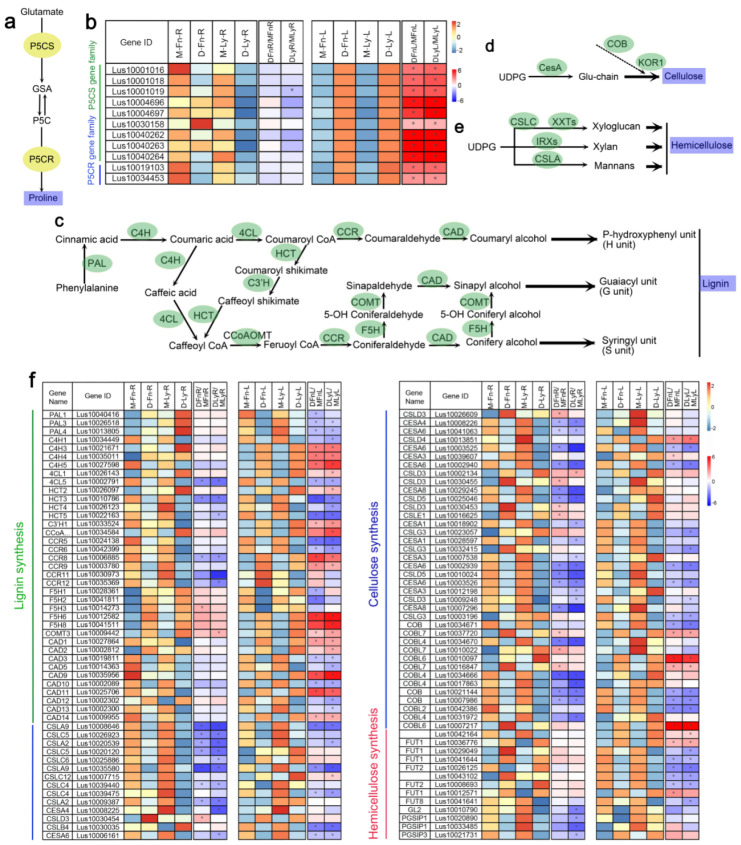
(**a**) The diagram of the proline biosynthesis pathway. (**b**) Expression profiles of genes related to proline synthesis in Fany and Longya10 flax under mock and drought stress conditions. (**c**) The gene expression is related to lignin synthesis. (**d**) The gene expression is related to cellulose synthesis. (**e**) The gene expression is related to hemicellulose synthesis. (**e**) Expression profiles of genes related to lignin-, cellulose- and hemicellulose-synthesis in Fany and Longya10 flax under mock and drought stress conditions. (**f**) A heatmap of DEGs is generated using the DESeq2 analysis. The abbreviations M-Fn and M-Ly represent flax growth in the mock condition derived from Fany and Longya10 flax, respectively. D-Fn and D-Ly represent flax growth in the stress condition derived from Fany and Longya10 flax. -R represents the root organ of flax, and -L represents the leaves organ of flax. The yellow color represents the expression levels of genes (in log2 transformed read counts per million), while the white color indicates the fold change (log2 value) of the DEGs. Statistical significance is denoted by asterisks (*) with *p* < 0.05.

In a previous report [15], it was stated that the expression of the *P5CS* gene family and *P5CR* gene family is associated with the drought stress process in flax. These genes are important in the proline biosynthesis pathway (Figure 6a). Observably, in our study, we observed higher expression levels of both *P5CS* and *P5CR* genes in the roots and lower expression levels in the leaves of flax (Figure 6b, Table S4), this upregulation of the *P5CS* and *P5CR* gene families in the leaves of flax indicated their significant involvement in response to drought stress.

Previous studies have reported on the genes involved in lignin biosynthesis, cellulose biosynthesis, and hemicellulose biosynthesis in plants [42]. In our study, we analyzed the lignin biosynthesis pathway accordingly (Figure 6c–e). The results showed that numerous genes were significantly regulated and induced by drought stress in flax (Figure 6f, Table S5). Most of the DEGs were downregulated following drought stress in flax. Furthermore, the number of DEGs in the roots was higher than that in the leaves following drought stress. The genes related to hemicellulose biosynthesis were significantly downregulated under drought stress in flax.

### 2.7. In Response to Drought Stress, the Expression of Phytohormone-Related Genes in Flax Was Significantly Altered

**Figure 7:**
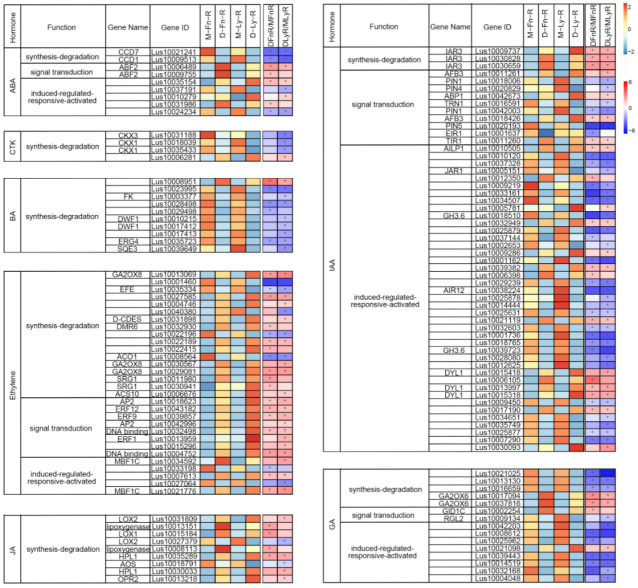
Expression profiles of genes related to phytohormones in roots of Fany and Longya10 flax under mock and drought stress conditions. A heatmap of DEGs is generated using the DESeq2 analysis. The abbreviations M-Fn and M-Ly represent flax growth in the mock condition derived from Fany and Longya10 flax, respectively. D-Fn and D-Ly represent flax growth in the stress condition derived from Fany and Longya10 flax. -R represents the root organ of flax, and -L represents the leaves organ of flax. The yellow color represents the expression levels of genes (in log2 transformed read counts per million), while the white color indicates the fold change (log2 value) of the DEGs. Statistical significance is denoted by asterisks (*) with *p* < 0.05.

We conducted an analysis of DEGs related to phytohormone gene expression variations induced by drought stress in the roots (Figure 7, Table S6) and leaves (Figure S2, Table S6) of flax. In the roots, we identified DEGs associated with abscisic acid (ABA) biosynthesis-degradation pathways, including Carotenoid Cleavage Dioxygenase 7 (*CCD7*) and *CCD1*, which were significantly downregulated following drought stress. The ABA signal-transduction gene Abscisic acid-responsive elements-Binging Factor 2 (*ABF2*) showed a considerable upregulation following drought stress. At the same time, DEGs related to cytokinin (CTK) biosynthesis-degradation, such as Cytokinin Oxidase3 (*CKX3*) and *CKX1*, were significantly downregulated in Longya10. Similarly, the BA biosynthesis-degradation gene Dwaf1 (*DWF1*) was downregulated in Longya10. Interestingly, most of the DEGs related to ethylene pathways were upregulated in response to drought stress in both Longya10 and Fany. These included ethylene biosynthesis-degradation genes such as Gibberellin 2-Oxidase 8 (*GA2OX8*), ACC synthase 10 (*ACS10*), and Senescence-Related Gene 1 (*SRG1*), as well as ethylene signal transduction genes like transcription factor AP2, Ethylene Response Factor 12 (*ERF12*), ERF9, ERF1, and ethylene-induced-regulated gene Multiprotein Bridging Factor 1C (*MBF1C*). Most of the DEGs related to jasmonic acid (JA) biosynthesis-degradation showed significant upregulation following drought stress. Moreover, the IAA biosynthesis-degradation gene IAA-Alanine Resistant 3 (*IAR3*) showed upregulation, and the IAA signal transduction gene Auxin Signaling F-box 3 (*AFB3*) was upregulated. In contrast, genes encoding auxin efflux carrier proteins, such as *PIN1* and *PIN4*, were down-regulated. IAA induced-regulated-responsive-activated auxin-responsive *GH3* family protein gene *GH3.6* was downregulated following drought stress in flax. Additionally, the results of phytohormones-related DEGs in the leaf tissue of flax were listed in Figure S2 (Table S6), which also exhibited extensive gene expression changes following drought stress in flax.

### 2.8. qRT-PCR Confirmed the Results of RNA-seq

**Figure 8:**
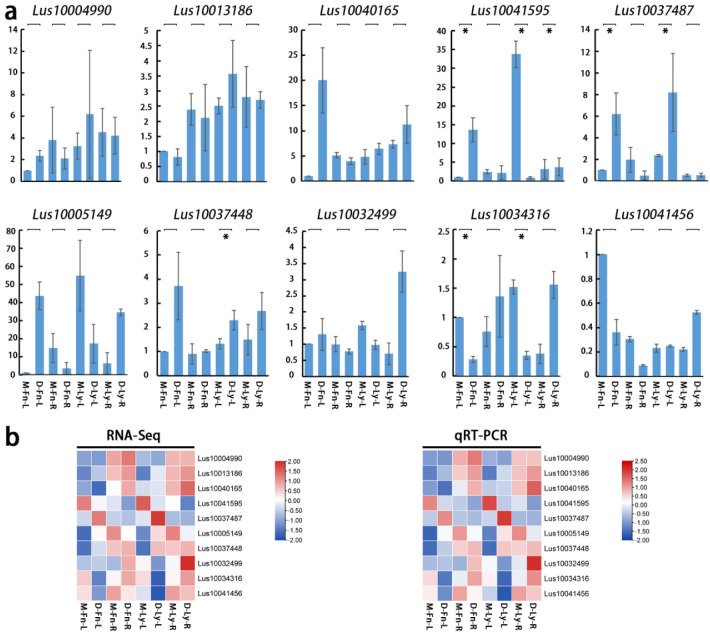
(**a**) The results of ten genes expression amplified by qRT-PCR. (**b**) The compare son results between RNA-seq and qRT-PCR. The abbreviations M-Fn and M-Ly represent flax growth in the mock condition derived from Fany and Longya10 flax, respectively. D-Fn and D-Ly represent flax growth in the stress condition derived from Fany and Longya10 flax. -R represents the root organ of flax, and -L represents the leaves organ of flax. Statistical significance is denoted by asterisks (*) with *p* < 0.05.

To further determine the accuracy of RNA-seq analysis for two varieties of flax under drought stress, we selected 10 genes randomly to amplified by qRT-PCR: *Lus10041595*, *Lus10037487*, *Lus10037448*, *Lus1003431*, *Lus10004990*, *Lus10013186*, *Lus10040165*, *Lus10005149*, *Lus10032499*, and *Lus10041456*. It was found that the expression levels of the selected 10 genes were consistent with those measured by RNA-seq (Figure 8).

## 3. Discussion

Drought stress has been identified as a significant factor affecting plant growth and development. Previous studies have reported that drought stress can lead to reduced photosynthesis [43], impaired cell elongation and division [44], and alterations in gene expression and yield [14,45,46]. In this study, we examined the effects of short-term drought stress on morphology, physiological characteristics, and gene expression in two flax varieties, Longya10 and Fany. Within 24 h, significant phenotypic changes were observed as a result of drought stress, including decreases in shoot weight, fresh weight, and water content. Additionally, various parameters related to stress response, such as proline and solute sugar content, MDA levels, CAT activity, SOD and POD activity, as well as lignin, acid-soluble lignin, acid-insoluble lignin, cellulose, and hemicellulose levels, showed rapid responses within the first 24 h of drought stress. We summarize these results to highlight some common stress responses exhibited by both oilseed flax and fiber flax in response to 24 h of drought (Figure S3). It has been reported that plants exhibit a range of biological responses when subjected to 24 h of water deficiency stress, which includes leaf wilting and curling, decreased relative water content, and show changes in physiological metabolism and antioxidant enzyme activity [47,48,49,50,51]. Similarly, in our study, the root length of flax did not show significant negative impacts following drought stress. The decline in RWC affects osmotic potential and reduces nutrient absorption in crops [52]. Studies conducted on wheat and rice have shown that water-stressed plants have lower water content compared to their well-watered counterparts, which can be associated with leaf tissue growth rate and transpiration rate [53,54]. In flax, the T-397 variety has been reported to exhibit moderate tolerance to drought stress, with the RWC of wilted shoots reaching 70% after four days and 60% after five days of drought stress [6]. In our study, we found that RWC in wilted seedlings was 90% and 85% in Fany and Longya10, respectively, within 24 h of drought stress. Notably, the RWL significantly increased in Longya10 following drought stress. Furthermore, water deprivation in plants leads to the generation of ROS, which reduces turgor pressure and causes oxidative damage [55]. In response to abiotic stress, various antioxidant enzymes and molecules, such as glutathione reductase (GTX), ascorbate peroxidase (APX), SOD, POD, proline, glycine betaine, soluble sugars, soluble proteins, and organic acids, are upregulated in the cytoplasm [56]. For instance, SOD converts elevated superoxide molecules into oxygen and H_2_O_2_, while POD converts H_2_O_2_ into oxygen and water [38], thus contributing to the defense against oxidative stress. Our experiments demonstrated that small molecular compounds accumulated in the roots of oil and fiber flax following drought stress, while variations in these compounds were relatively minor in the shoots (Figure 2). These findings suggest that significant biochemical responses occur in the roots of flax following short-term drought stress, particularly in the Fany variety. Additionally, the decline in turgor pressure negatively affects plant cell development under drought stress [24]. Specifically, reduced water availability from the xylem to neighboring cells can impact cell elongation [57]. Our results indicated significant accumulations of lignin and acid-soluble lignin in the roots, stems, and leaves of Fany following drought stress (Figure 3), suggesting that cell development, as a physiological process, is more sensitive to drought stress in fiber flax compared to oil flax.

The observed variations in morphological, physiological, and biochemical changes in plants under drought stress are regulated by critical gene expression [58]. Transcriptome analysis revealed significant alterations in genome-wide gene expression across the flax genome following short-term drought stress in our study. Many DEGs exhibited specificity in their response to drought stress, tissue/organ specificity, and genotype specificity when comparing stressed plants with their respective controls in Fany and Longya10 (Figure 4). Additionally, stresses-induced DEGs in flax were performing GO and KEGG enrichment analyses. It has been observed that oxidative stress increases and leads to a reduction in chlorophyll II concentration in plants under drought stress [59]. Consequently, the disturbance in plant growth and development due to reduced photosynthesis rates is associated with drought stress [60]. GO analysis results demonstrated that stressed DEGs were enriched in photosynthesis (GO:0015979), photosynthesis-light reaction (GO:0019684), and photosynthesis-light harvesting (GO:0009765) (Figure 5a). KEGG analysis revealed that stressed DEGs were enriched in photosynthesis, photosynthesis-antenna protein, and photosynthesis (Figure 5b). The genes associated with GO:0015979, GO:0019684, and GO:0009765 are listed in Figure 5c,d. These findings indicate that all the identified genes were downregulated in flax leaves but upregulated in flax roots following short-term drought stress. Our experiments demonstrated that drought stress-regulated the expression of genes related to photosynthesis, leading to a downregulation of photosynthesis in flax leaves. Additionally, previous studies have shown that lipids, as the main components of cell membranes, can trigger signaling cascades under abiotic stress and activate plant responses [61]. We observed a significant enrichment of lipid metabolism in flax leaves following drought stress. Moreover, fatty acid degradation was notably increased in the leaves of Longya10 after drought stress, suggesting that short-term drought stress may affect fatty acid content in oil flax, which warrants further investigation.

It had been reported that overexpression of the member of the *P5CS* gene family caused the content of proline raised, which enhanced the drought stress tolerance in plants [17]. Similarly, overexpression of *P5CR* genes has been shown to enhance the photosynthetic response under drought and high-temperature stress in *Arabidopsis* [18,19]. In a previous report [15], members of the *P5CS* and *P5CR* gene families were identified and found to be upregulated by drought stress in the Z141 variety of flax. Consistent with these findings, we observed significant upregulation of genes involved in proline biosynthesis pathways in the leaves of Fany and Longya10 following short-term drought stress in our experiments (Figure 6b). This suggests a differential response in the expression of proline biosynthesis-related genes between the leaves and roots of flax following drought stress. Despite the upregulation of proline biosynthesis-related gene expression, the proline content increased in the leaves of Longya10 but decreased in the leaves of Fany after drought stress. These findings indicate that there may not be a direct correlation between the level of proline and the expression of *P5CS*/*P5CR* genes in flax under drought stress.

In response to water insufficiency, the upregulation of lignification serves as a strategy for plants to adapt to stressful environments. However, understanding the lignification response during drought stress has been challenging due to variations based on species, organs/tissues, and the intensity and duration of the stress period [62]. Previous studies have identified genes involved in lignin biosynthesis in flax [25], and we examined these genes in our study (Figure 6f). Consistent with previous findings [25], our results demonstrated that most of the related gene expression levels were higher in roots compared to leaves. Which indicated that the changes in gene expression were more pronounced in leaves compared to roots. The lignin biosynthesis-DEGs results indicated that genes involved in the same biological process were co-regulated in oil and fiber flax following drought. Previous research has reported that genes such as *LusC4H1*, *LusC4H3*, *LusCCR4*, *LusCCR8*, *LusCCR10*, and *LusCAD11* were upregulated in flax leaves after 12 days of drought stress [25]. In our study, we observed the upregulation of several genes, including *LusC4H1* and *LusCCR10*, following short-term drought stress in the leaves of Fany and Longya10, providing additional support for the expression of lignification-related genes during drought stress. Flax, being an economically important species rich in cellulose and hemicellulose, particularly in its bast fibers, was also examined. By inputting the stressed DEGs into the MapMan software (Version 3.6.0), we identified genes related to cellulose and hemicellulose, although their expression levels were lower compared to lignin biosynthesis-related genes. Most of these DEGs were downregulated in both leaves and roots of flax under short-term drought stress. These findings further support the regulation of cytoderm composition-related gene expression during drought stress.

Plant hormones play crucial roles in regulating plant growth and development in different environments [36,37]. It has been established that abscisic acid (ABA), gibberellins, and cytokinins function as plant growth regulators in response to water stress [63]. Phytohormones also mediate internal and external stimuli and signal transduction pathways [42]. To investigate the genes involved in plant hormone metabolism, we inputted the drought-stressed DEGs into the MapMan software. We analyzed genes related to phytohormone metabolism in the roots and leaves of Fany and Longya10, respectively (Figure 7 and Figure S2, Table S6). The *ABFs* genes, known to be involved in stomatal closure mediated by ABA in response to osmotic stress in plants [64], showed higher expression levels in the roots of flax after drought stress, supporting their role in response to drought. *CCD1* and *CCD7*, genes involved in strigolactone (SL) biosynthesis, exhibited lower expression in the roots of flax after drought stress. Cytokinin oxidase/dehydrogenases (CKXs) are key enzymes involved in cytokinin (CTK) degradation. It has been reported that *OsCKX2* negatively regulates Pi deficiency tolerance by modulating CTK levels in rice [65]. In our study, the genes *CKX1* and *CKX3* showed decreased expression in the roots of Longya10 after drought stress. Ethylene levels increase in response to abiotic stress in plants [66], and AP2/ERF transcription factors are involved in ethylene signaling pathways, exhibiting expression changes induced by saline stress in rice [47]. In our study, the genes *AP2*, *ERF1*, *ERF9*, and *ERF12* showed increased expression in the roots of flax after drought stress, supporting the role of transcription factors in receiving upstream signals and regulating the expression of downstream resistance genes [38]. Most of the jasmonic acid (JA) biosynthesis genes, such as LOXs, were upregulated in response to saline stress in rice [47]. In our study, the gene *LOX1* showed significant upregulation in the roots of Fany after drought stress. Most IAA/GA-related genes were downregulated in the roots of Fany and Longya10 after drought stress. Based on the gene expression variations observed in flax following short-term drought stress, phytohormones appear to function as essential signaling molecules that regulate flax development and growth under drought stress conditions.

This study aimed to investigate the variations in morphology, physiology, biochemistry, and gene expression in the leaves and roots of Fany and Longya10 flax varieties under short-term drought stress. The morphology of flax leaves was significantly affected by drought stress. Furthermore, we observed rapid increases in the levels of small organic compounds in response to drought stress in flax. Within 12 h of drought stress, the expression of genes related to photosynthesis, lignin/cellulose/hemicellulose biosynthesis, and phytohormone metabolism was altered. These findings contribute valuable insights into the response of flax to abiotic stress and establish a foundation for further investigations in this field.

## 4. Methods and Materials

### 4.1. The Treatment of Drought Stress

To investigate the genome-wide variation resulting from short-term drought stress in oil and fiber flax, two flax varieties, Longya10 and Fany, were selected for the experiment. The seeds of these varieties were washed with distilled water and planted in a mixed soil medium (vermiculite: soil 1:1). The plants were then placed in an artificial illumination incubator with a temperature regime of 26 °C/18 °C and a photoperiod of 16 h light and 8 h dark. After 18 days of growth, when the flax seedlings reached a height of approximately 10 cm, they were carefully removed from the soil and rinsed. These seedlings were then subjected to drought stress in 200 mL conical flasks. Plant dehydration stress was induced using a PEG solution, following a method described in a previous report [41]. The PEG solution had concentrations of 0%, 10%, 15%, and 20%, and the flax seedlings were treated with these solutions for 24 h. The control group was treated with distilled water (0% PEG solution). Based on pretest results, a 10% PEG solution was chosen for the drought stress treatment. Both stressed, and mock (control) flax seedlings of Longya10 and Fany were harvested for various measurements, including fresh seedling weight, shoot fresh weight, and root fresh weight, following the drought stress treatment. Each biological replicate consisted of three technical replicates, and for each biological replicate, ten plants were mixed together. The relative water content and the rate of water loss were determined using methods described by Ghashghaie [67] and Li [68], respectively. Additionally, various organs of the flax plants were harvested for analysis of small organic compounds, enzyme activity, and transcriptome profiling. Similarly, parallel plants grown under mock conditions were harvested at the same time as the control group. To facilitate sample identification, a naming pattern was adopted for all the samples. The samples were named following the pattern “mock/stress-genotype-organ.” For example, the root, stem, and leaf samples for Longya10 under mock conditions were named M-Ly-R, M-Ly-S, and M-Ly-L, respectively. Similarly, the corresponding samples under drought stress were named D-Ly-R, D-Ly-S, and D-Ly-L. The same naming convention was applied to the samples from the Fany variety (M-Fn-R, M-Fn-S, M-Fn-L, D-Fn-R, D-Fn-S, and D-Fn-L).

### 4.2. The Small Organic Compounds and Enzyme Activity Detection

To determine the levels of small organic compounds and enzyme activity, several parameters were measured after subjecting the samples to 24 h of drought stress. The measurements included proline, soluble protein, soluble sugar, MDA, CAT, SOD, and POD. The measurements were performed using a SmartSpecTM Plus spectrophotometer (BioRad, Hercules, CA, USA). Approximately 0.1 g of dried root or leaf samples was extracted using 80% ethanol. Activated carbon was added to the centrifuge, and the supernatants were transferred and filtered for further analysis of proline and soluble sugar content. The filtrate was treated with the ninhydrin reagent solution, and the resulting color was measured at a wavelength of 520 nm. The proline content was determined using a standard curve, with the color measured at a wavelength of 625 nm. Similarly, the soluble sugar content was determined using a standard curve, as described in a previous report [69]. The measurement of the soluble protein involved the binding of Coomassie Brilliant Blue G-250 to the protein samples, followed by colorimetric analysis at a wavelength of 595 nm, following a method described in a previous report [70]. For the measurement of MDA, approximately 0.5 g of tissues were ground under liquid nitrogen and placed into a phosphate buffer (50 mM, pH 7.8). The mixture was centrifuged for 10 min, and the supernatant was transferred for further analysis of MDA content, following a method described in a previous report [70]. CAT activity was assessed by monitoring the decline in absorbance per minute at 240 nm, resulting from the reaction between potassium phosphate buffer and H_2_O_2_, according to a method described in a previous report [70]. SOD activity was measured based on the photochemical reduction of NBT (nitroblue tetrazolium), while POD activity was measured based on the absorbance induced by guaiacol. The measurement methods for both SOD and POD activity were described in a previous report [70]. Take 0.1 g tissue to join containing 1% polyvinylpyrrolidone 50 mm phosphate buffer (pH 7.8) after grinding homogenate, centrifuge the mixture solution for 20 min, transfer the supernatant and to manage the solution then assayed by 560 nm and 470 nm absorbance, respectively.

### 4.3. The Lignin and Cellulose Contents Detection

To determine the contents of lignin, cellulose, and hemicellulose, the SmartSpecTM Plus spectrophotometer (BioRad, Hercules, CA, USA) was utilized after subjecting the samples to 24 h of drought stress. For the measurement of lignin content, approximately 15 mg of flax tissues was ground and heated at 100 °C for 2 h. The ground tissues were then mixed with water and incubated at 65 °C for 1 h. The mixture was filtered using a GF/A glass fiber filter and rinsed sequentially with water, ethanol, acetone, and diethyl ether at 70 °C overnight. Subsequently, the filtered sample was mixed with acetic acid (25% acetyl bromide) and incubated at 50 °C for 2 h. Sodium hydroxide (2 N) and acetic acid were added to the mixture, which was then incubated overnight. The solution was measured at a wavelength of 280 nm, and the lignin content was determined using a standard curve, following a method described in a previous report [71]. For the measurement of cellulose content, approximately 15 mg of tissues were ground and mixed with a solution of nitric and acetic acid (1:8 *v*/*v*). After 1 h of incubation, the sample was heated at 100 °C, and the supernatant was removed following centrifugation. The pellet was washed, resuspended, and treated with an anthrone reagent solution. The resulting color was measured at a wavelength of 620 nm, and the cellulose content was determined using a standard curve, following a method described in a previous report [72].

### 4.4. The RNA Isolation and RNA Sequencing (RNA-seq)

After subjecting Longya10 and Fany to 10% PEG solution treatment for 24 h, the roots, and leaves of these plants were harvested. At the same time, parallel plants grown under mock conditions were also harvested as controls. In total, eight samples were collected for transcriptomic analysis. To isolate total RNA, the Trizol Reagent (Life Technologies Invitrogen, Carlsbad, CA, USA) was used following the manufacturer’s instructions. The RNA was then treated with RNase-free DNase I (Life Technologies Invitrogen, Carlsbad, CA, USA) to eliminate possible genomic DNA contamination before being reverse-transcribed with the SuperScript RNase H- Reverse Transcriptase (Life Technologies Invitrogen, Carlsbad, CA, USA).

The total RNA was prepared for RNA sequencing based on the Illumina Sample Preparation Protocol (Illumina, San Diego, CA, USA). The Novaseq platform was used to generate sequencing data, with 125 bp paired-end reads and quality thresholds of Q20 > 95% and Q30 > 88%. Each sample obtained approximately 30–40 million raw sequences (Table S1). The raw sequences were processed by removing adaptors, low-quality sequences, and poly-A sequences, resulting in clean reads. To map the clean reads to the reference genome [41], BWA and STAR algorithms were employed, following a method described in a previous report [47]. DESeq2 R package was used for data normalization and calculation of DEGs with the criteria of a max_readcount > 30, fold change > 2, and *p* < 0.05. A total of 24 libraries were constructed, representing three biological replicates for each sample. The clean data has been deposited in the SRA database (http://www.ncbi.nlm.nih.gov/sra/, accessed on 31 May 2023) with accession numbers PRJNA977728.

### 4.5. The GO Classification and KEGG Classification Analysis

Molecular function category GO terms was analyzed by the online agriGOv2 platform (http://systemsbiology.cau.edu.cn/agriGOv2/index.php, accessed on 15 March 2022) with FDR corrected *p*-value < 0.05 (Fisher tests). In addition, pathway assignments were performed following the Kyoto Encyclopedia of Genes and Genomes (KEGG)mapping (http://www.genome.ad.jp/kegg/kegg2.html, accessed on 15 March 2022) as our previous report [73].

### 4.6. qRT-PCR Validation

To validate the RNA sequencing data, a quantitative real-time polymerase chain reaction (qRT-PCR) was performed. Ten DEGs were randomly selected for analysis, following a methodology similar to a previous report [47]. The qRT-PCR reactions were carried out using the SYBR Green I PCR master mix kit (TaKaRa, Tokyo, Japan) and were repeated three times, following the procedures described in the previous report [47]. Gene-specific primer pairs were obtained from qPrimerDB (https://biodb.swu.edu.cn/qprimerdb/, accessed on 10 Janarary 2022, Table S7). The results of the qRT-PCR analysis are presented in Figure 8.

### 4.7. Statistical Analysis

We summarised the results of physiological and biochemical indices and lignin and input them into the DPS data processing system to obtain the results of analysis of variance (ANOVA) of the experimental groups of different flax tissues and calculated parameters such as mean, variance, and coefficient of variation for the comparison of Longya 10 and Fany [74]. After that, the use of RStudio software (Version 1.4) for statistical analysis of physiological and biochemical indicators and lignin and other results, to Pearson’s correlation to estimate their associations, and finally, *p* < 0.05 as the criterion and correlation with the corrplot package to calculate the significance of the correlation (https://github.com/taiyun/corrplot, accessed on 16 Janarary 2022). All statistical analyses were performed with t-tests and presented as the mean ± standard error of three biological replicates. The threshold for statistical significance was set at *p* < 0.05.

## Data Availability

The datasets generated and analyzed in this study are available at [PRJNA977728] https://www.ncbi.nlm.nih.gov/sra/PRJNA977728, accessed on 31 May 2023.

## Supplementary Materials

The supporting information can be downloaded at: https://www.mdpi.com/article/10.3390/ijms241512019/s1.

## References

[1] Dash J., Naik B., Mohapatra U. (2017). Linseed: A Valuable Crop Plant. Int. J. Adv. Res..

[2] Duigou A., Baley C. (2012). Flax and Hemp Fibres: A Natural Solution for the Composite Industry, Eco Design, Life Cycle Analysis and Recycling.

[3] Zhang J., Dang Z., She X., Zhao L., Wang L. (2009). Breeding of new oil flax cultivar Longya No 10 with high yields good quality and resistance to diseases. Agric. Res. Arid. Areas.

[4] Prasad K. (2014). Flaxseed and cardiovascular health. J. Cardiovasc. Pharmacol..

[5] Gill K.S. (1987). Flax.

[6] Dash P.K., Cao Y., Jailani A.K., Gupta P., Venglat P., Xiang D., Rai R., Sharma R., Thirunavukkarasu N., Abdin M.Z. (2014). Genome-wide analysis of drought induced gene expression changes in flax (*Linum usitatissimum*). GM Crops Food.

[7] Araus J. (2002). Plant Breeding and Drought in C3 Cereals: What Should We Breed For?. Ann. Bot..

[8] Hu H., Xiong L. (2014). Genetic Engineering and Breeding of Drought-Resistant Crops. Annu. Rev. Plant Biol..

[9] Urano K., Maruyama K., Jikumaru Y., Kamiya Y., Yamaguchi-Shinozaki K., Shinozaki K. (2017). Analysis of plant hormone profiles in response to moderate dehydration stress. Plant J..

[10] Yang C., Liu J., Dong X., Cai Z., Tian W., Wang X. (2014). Short-term and continuing stresses differentially interplay with multiple hormones to regulate plant survival and growth. Mol. Plant.

[11] Li W., Li Q. (2017). Effect of Environmental Salt Stress on Plants and the Molecular Mechanism of Salt Stress Tolerance. Int. J. Environ. Sci. Nat. Resour..

[12] Tripathy J.N., Zhang J., Robin S., Nguyen T.T., Nguyen H.T. (2000). QTLs for cell-membrane stability mapped in rice (*Oryza sativa* L.) under drought stress. Theor. Appl. Genet..

[13] Abreha K.B., Enyew M., Carlsson A.S., Vetukuri R.R., Feyissa T., Motlhaodi T., Ng’uni D., Geleta M. (2021). Sorghum in dryland: Morphological, physiological, and molecular responses of sorghum under drought stress. Planta.

[14] Wu J., Zhao Q., Sun D., Wu G., Zhang L., Yuan H., Yu Y., Zhang S., Yang X., Li Z. (2018). Transcriptome analysis of flax (*Linum usitatissimum* L.) undergoing osmotic stress. Ind. Crops Prod..

[15] Wang W., Wang L., Wang L., Tan M., Ogutu C.O., Yin Z., Zhou J., Wang J., Wang L., Yan X. (2021). Transcriptome analysis and molecular mechanism of linseed (*Linum usitatissimum* L.) drought tolerance under repeated drought using single-molecule long-read sequencing. BMC Genom..

[16] Sun J., He L., Li T. (2019). Response of seedling growth and physiology of *Sorghum bicolor* (L.) Moench to saline-alkali stress. PLoS ONE.

[17] Maghsoudi K., Emam Y., Niazi A., Pessarakli M., Arvin M.J. (2018). P5CS expression level and proline accumulation in the sensitive and tolerant wheat cultivars under control and drought stress conditions in the presence/absence of silicon and salicylic acid. J. Plant Interact..

[18] De Ronde J.A., Cress W.A., Kruger G.H., Strasser R.J., Van Staden J. (2004). Photosynthetic response of transgenic soybean plants, containing an Arabidopsis P5CR gene, during heat and drought stress. J. Plant Physiol..

[19] Benitez L.C., Vighi I.L., Auler P.A., do Amaral M.N., Moraes G.P., dos Santos Rodrigues G., da Maia L.C., de Magalhães Júnior A.M., Braga E.J.B. (2016). Correlation of proline content and gene expression involved in the metabolism of this amino acid under abiotic stress. Acta Physiol. Plant..

[20] Jones L., McQueen-Mason S. (2004). A role for expansins in dehydration and rehydration of the resurrection plant Craterostigma plantagineum. FEBS Lett..

[21] Xu L., Naylor D., Dong Z., Simmons T., Pierroz G., Hixson K.K., Kim Y.M., Zink E.M., Engbrecht K.M., Wang Y. (2018). Drought delays development of the sorghum root microbiome and enriches for monoderm bacteria. Proc. Natl. Acad. Sci. USA.

[22] Kim Y., Chung Y.S., Lee E., Tripathi P., Heo S., Kim K.H. (2020). Root Response to Drought Stress in Rice (*Oryza sativa* L.). Int. J. Mol. Sci..

[23] Kosakivska I.V., Vedenicheva N.P., Babenko L.M., Voytenko L.V., Romanenko K.O., Vasyuk V.A. (2022). Exogenous phytohormones in the regulation of growth and development of cereals under abiotic stresses. Mol. Biol. Rep..

[24] Elansary H.O., Abdel-Hamid A.M.E., Yessoufou K., Al-Mana F.A., El-Ansary D.O., Mahmoud E.A., Al-Yafrasi M.A. (2020). Physiological and molecular characterization of water-stressed *Chrysanthemum* under robinin and chitosan treatment. Acta Physiol. Plant..

[25] Le Roy J., Blervacq A.S., Creach A., Huss B., Hawkins S., Neutelings G. (2017). Spatial regulation of monolignol biosynthesis and laccase genes control developmental and stress-related lignin in flax. BMC Plant Biol..

[26] Gigli-Bisceglia N., Engelsdorf T., Hamann T. (2020). Plant cell wall integrity maintenance in model plants and crop species-relevant cell wall components and underlying guiding principles. Cell. Mol. Life Sci..

[27] Chantreau M., Chabbert B., Billiard S., Hawkins S., Neutelings G. (2015). Functional analyses of cellulose synthase genes in flax (*Linum usitatissimum*) by virus-induced gene silencing. Plant Biotechnol. J..

[28] Chabi M., Goulas E., Leclercq C.C., de Waele I., Rihouey C., Cenci U., Day A., Blervacq A.S., Neutelings G., Duponchel L. (2017). A Cell Wall Proteome and Targeted Cell Wall Analyses Provide Novel Information on Hemicellulose Metabolism in Flax. Mol. Cell. Proteom..

[29] Dwivany F.M., Yulia D., Burton R.A., Shirley N.J., Wilson S.M., Fincher G.B., Bacic A., Newbigin E., Doblin M.S. (2009). The CELLULOSE-SYNTHASE LIKE C (CSLC) family of barley includes members that are integral membrane proteins targeted to the plasma membrane. Mol. Plant.

[30] Zabotina O.A., van de Ven W.T., Freshour G., Drakakaki G., Cavalier D., Mouille G., Hahn M.G., Keegstra K., Raikhel N.V. (2008). Arabidopsis XXT5 gene encodes a putative alpha-1,6-xylosyltransferase that is involved in xyloglucan biosynthesis. Plant J..

[31] Ullah A., Manghwar H., Shaban M., Khan A.H., Akbar A., Ali U., Ali E., Fahad S. (2018). Phytohormones enhanced drought tolerance in plants: A coping strategy. Environ. Sci. Pollut. Res..

[32] Niu M., Xie J., Chen C., Cao H., Sun J., Kong Q., Shabala S., Shabala L., Huang Y., Bie Z. (2018). An early ABA-induced stomatal closure, Na^+^ sequestration in leaf vein and K^+^ retention in mesophyll confer salt tissue tolerance in Cucurbita species. J. Exp. Bot..

[33] Chen K., Li G.J., Bressan R.A., Song C.P., Zhu J.K., Zhao Y. (2020). Abscisic acid dynamics, signaling, and functions in plants. J. Integr. Plant Biol..

[34] Yang S., Yu Q., Zhang Y., Jia Y., Wan S., Kong X., Ding Z. (2018). ROS: The Fine-Tuner of Plant Stem Cell Fate. Trends Plant Sci..

[35] Han J.P., Koster P., Drerup M.M., Scholz M., Li S., Edel K.H., Hashimoto K., Kuchitsu K., Hippler M., Kudla J. (2019). Fine-tuning of RBOHF activity is achieved by differential phosphorylation and Ca^2+^ binding. New Phytol..

[36] Yu Z., Duan X., Luo L., Dai S., Ding Z., Xia G. (2020). How Plant Hormones Mediate Salt Stress Responses. Trends Plant Sci..

[37] Singh P., Dutta P., Chakrabarty D. (2021). miRNAs play critical roles in response to abiotic stress by modulating cross-talk of phytohormone signaling. Plant Cell Rep..

[38] Fang S., Hou X., Liang X. (2021). Response Mechanisms of Plants Under Saline-Alkali Stress. Front. Plant Sci..

[39] Wondimu B., Rethman N.F.G., Hammes P.S., Pieterse P.A., Grimbeek J., Linde M.V.D. (2005). Water stress affects the germination, emergence and growth of different sorghum cultivars. SINET Ethiop. J. Sci..

[40] Queiroz M.S., Oliveira C.E.S., Steiner F., Zuffo A.M., Zoz T., Vendruscolo E.P., Silva M.V., Mello B.F.F.R., Cabral R.C., Menis F.T. (2019). Drought Stresses on Seed Germination and Early Growth of Maize and Sorghum. J. Agric. Sci..

[41] Wang N., Lin Y., Qi F., Xiaoyang C., Peng Z., Yu Y., Liu Y., Zhang J., Qi X., Deyholos M. (2022). Comprehensive Analysis of Differentially Expressed Genes and Epigenetic Modification-Related Expression Variation Induced by Saline Stress at Seedling Stage in Fiber and Oil Flax, *Linum usitatissimum* L. Plants.

[42] Wahab A., Abdi G., Saleem M.H., Ali B., Ullah S., Shah W., Mumtaz S., Yasin G., Muresan C.C., Marc R.A. (2022). Plants’ Physio-Biochemical and Phyto-Hormonal Responses to Alleviate the Adverse Effects of Drought Stress: A Comprehensive Review. Plants.

[43] McKay J.K., Richards J.H., Mitchell-Olds T. (2003). Genetics of drought adaptation in *Arabidopsis thaliana*: I. Pleiotropy contributes to genetic correlations among ecological traits. Mol. Ecol..

[44] Hussain M., Malik M.A., Farooq M., Ashraf M.Y., Cheema M.A. (2008). Improving Drought Tolerance by Exogenous Application of Glycinebetaine and Salicylic Acid in Sunflower. J. Agron. Crop Sci..

[45] Cattivelli L., Rizza F., Badeck F.-W., Mazzucotelli E., Mastrangelo A.M., Francia E., Marè C., Tondelli A., Stanca A.M. (2008). Drought tolerance improvement in crop plants: An integrated view from breeding to genomics. Field Crops Res..

[46] Kumar M., Kumar Patel M., Kumar N., Bajpai A.B., Siddique K.H.M. (2021). Metabolomics and Molecular Approaches Reveal Drought Stress Tolerance in Plants. Int. J. Mol. Sci..

[47] Wang N., Fan X., Lin Y., Li Z., Wang Y., Zhou Y., Meng W., Peng Z., Zhang C., Ma J. (2022). Alkaline Stress Induces Different Physiological, Hormonal and Gene Expression Responses in Diploid and Autotetraploid Rice. Int. J. Mol. Sci..

[48] Wang N., Wang S., Qi F., Wang Y., Lin Y., Zhou Y., Meng W., Zhang C., Wang Y., Ma J. (2022). Autotetraploidization Gives Rise to Differential Gene Expression in Response to Saline Stress in Rice. Plants.

[49] Yang Y., Mo Y., Yang X., Zhang H., Wang Y., Li H., Wei C., Zhang X. (2016). Transcriptome Profiling of Watermelon Root in Response to Short-Term Osmotic Stress. PLoS ONE.

[50] Fan H.-F., Ding L., Du C.-X., Wu X. (2014). Effect of short-term water deficit stress on antioxidative systems in cucumber seedling roots. Bot. Stud..

[51] Dudziak K., Zapalska M., Borner A., Szczerba H., Kowalczyk K., Nowak M. (2019). Analysis of wheat gene expression related to the oxidative stress response and signal transduction under short-term osmotic stress. Sci. Rep..

[52] Ashrafi M., Azimi-Moqadam M.R., MohseniFard E., Shekari F., Jafary H., Moradi P., Pucci M., Abate G., Mastinu A. (2022). Physiological and Molecular Aspects of Two Thymus Species Differently Sensitive to Drought Stress. BioTech.

[53] Kapoor D., Bhardwaj S., Landi M., Sharma A., Ramakrishnan M., Sharma A. (2020). The Impact of Drought in Plant Metabolism: How to Exploit Tolerance Mechanisms to Increase Crop Production. Appl. Sci..

[54] Yan F., Sun Y., Xu H., Yin Y., Wang H., Wang C., Guo C., Yang Z., Sun Y., Ma J. (2018). Effects of wheat straw mulch application and nitrogen management on rice root growth, dry matter accumulation and rice quality in soils of different fertility. Paddy Water Environ..

[55] Alam H., Khattak J.Z.K., Ksiksi T.S., Saleem M.H., Fahad S., Sohail H., Ali Q., Zamin M., El-Esawi M.A., Saud S. (2021). Negative impact of long-term exposure of salinity and drought stress on native *Tetraena mandavillei* L. Physiol. Plant..

[56] Raza A., Mehmood S.S., Tabassum J., Batool R. (2019). Targeting Plant Hormones to Develop Abiotic Stress Resistance in Wheat. Wheat Production in Changing Environments.

[57] Ali Q., Shahid S., Nazar N., Hussain A.I., Ali S., Chatha S.A.S., Perveen R., Naseem J., Haider M.Z., Hussain B., Hasanuzzaman M. (2020). Use of Phytohormones in Conferring Tolerance to Environmental Stress. Plant Ecophysiology and Adaptation under Climate Change: Mechanisms and Perspectives II: Mechanisms of Adaptation and Stress Amelioration.

[58] Singh B., Norvell E., Wijewardana C., Wallace T., Chastain D., Reddy K.R. (2018). Assessing morphological characteristics of elite cotton lines from different breeding programmes for low temperature and drought tolerance. J. Agron. Crop Sci..

[59] Allakhverdiev S.I. (2020). Optimising photosynthesis for environmental fitness. Funct. Plant Biol..

[60] Ferrara A., Lovelli S., Tommaso T.D., Perniola M. (2011). Flowering, Growth and Fruit Setting in Greenhouse Bell Pepper under Water Stress. J. Agron..

[61] Hou Q., Ufer G., Bartels D. (2016). Lipid signalling in plant responses to abiotic stress. Plant Cell Environ..

[62] Cabane M., Afif D., Hawkins S. (2012). Lignins and Abiotic Stresses. Lignins—Biosynthesis, Biodegradation and Bioengineering.

[63] Chen K., Wang Y., Zhang R., Zhang H., Gao C. (2019). CRISPR/Cas Genome Editing and Precision Plant Breeding in Agriculture. Annu. Rev. Plant Biol..

[64] Cai S., Chen G., Wang Y., Huang Y., Marchant D.B., Wang Y., Yang Q., Dai F., Hills A., Franks P.J. (2017). Evolutionary Conservation of ABA Signaling for Stomatal Closure. Plant Physiol..

[65] Yan H., Wang Y., Chen B., Wang W., Sun H., Sun H., Li J., Zhao Q. (2022). OsCKX2 regulates phosphate deficiency tolerance by modulating cytokinin in rice. Plant Sci..

[66] Waadt R., Seller C.A., Hsu P.-K., Takahashi Y., Munemasa S., Schroeder J.I. (2022). Plant hormone regulation of abiotic stress responses. Nat. Rev. Mol. Cell Biol..

[67] Ghashghaie J., Brenckmann F., Saugier B. (1992). Water relations and growth of rose plants cultured in vitro under various relative humidities. Plant Cell Tissue Organ Cult..

[68] Zhao X., Bai S., Li L., Han X., Li J., Zhu Y., Fang Y., Zhang D., Li S. (2020). Comparative Transcriptome Analysis of Two Aegilops tauschii with Contrasting Drought Tolerance by RNA-Seq. Int. J. Mol. Sci..

[69] Li Q., Yang A., Zhang W.H. (2017). Comparative studies on tolerance of rice genotypes differing in their tolerance to moderate salt stress. BMC Plant Biol..

[70] Liu D., Liu M., Liu X.L., Cheng X.G., Liang Z.W. (2018). Silicon Priming Created an Enhanced Tolerance in Alfalfa (*Medicago sativa* L.) Seedlings in Response to High Alkaline Stress. Front. Plant Sci..

[71] Menezes-Silva P.E., Sanglard L., Avila R.T., Morais L.E., Martins S.C.V., Nobres P., Patreze C.M., Ferreira M.A., Araujo W.L., Fernie A.R. (2017). Photosynthetic and metabolic acclimation to repeated drought events play key roles in drought tolerance in coffee. J. Exp. Bot..

[72] Tombesi S., Frioni T., Poni S., Palliotti A. (2018). Effect of water stress “memory” on plant behavior during subsequent drought stress. Environ. Exp. Bot..

[73] Lin Y., Ma J., Wu N., Qi F., Peng Z., Nie D., Yao R., Qi X., Slaski J., Yang F. (2022). Transcriptome Study of Rice Roots Status under High Alkaline Stress at Seedling Stage. Agronomy.

[74] Wei T., Simko V., Levy M.X.Y., Jin Y., Zemla J. (2017). Package: Corrplot. Visualization of a Correlation Matrix, Version: 0.84. Statistician.

