# Supplementary material for: Evaluation of Differentially Expressed Genes in Leaves vs. Roots Subjected to Drought Stress in Flax (Linum usitatissimum L.)
Source: Int J Mol Sci. 2023 Jul 27;24(15):12019. doi: 10.3390/ijms241512019 (PMC10419004; doi:10.3390/ijms241512019)
Supplement: Supplementary file 1 [file ijms-24-12019-s001.zip › supplementary materials captions.pdf]

Figure S1. DEGs were characterized in roots and leaves of Fany and Longya10 flax plants under simulated (control) and drought stress conditions. (a) DEGs analyzed for different flax controls versus drought stress induced were identified and classified as up-regulated (red) and down-regulated (blue). (b) DEGs were analyzed to determine that they are specifically induced in certain organs or tissues of flax. (c) DEGs analyzed to determine that they are specifically induced in certain varieties or species of flax. Details of DEGs between all samples were included in the Venn analysis, which helped to identify common and unique DEGs between different conditions, organs and flax species.

Figure S2: Expression profiles of genes related to phytohormones in leaves of Fany and Longya10 flax under mock and drought stress conditions.

Figure S3: A picture illustrates how flax responds to drought condition in this study.

Table S1: The overview of RNA-seq data.

Table S2: The data of GO analysis.

Table S3: The data of KEGG analysis.

Table S4: The data of photosynthesis and proline related genes expression analysis.

Table S5: The data of lignin and cellulose related genes expression analysis.

Table S6: The data of phytohormones related genes expression analysis.

Table S7: The list of primers for qRT-PCR.
