# Supplementary figures and images for: Evaluation of Differentially Expressed Genes in Leaves vs. Roots Subjected to Drought Stress in Flax (Linum usitatissimum L.)
Source: Int J Mol Sci. 2023 Jul 27;24(15):12019. doi: 10.3390/ijms241512019 (PMC10419004; doi:10.3390/ijms241512019)

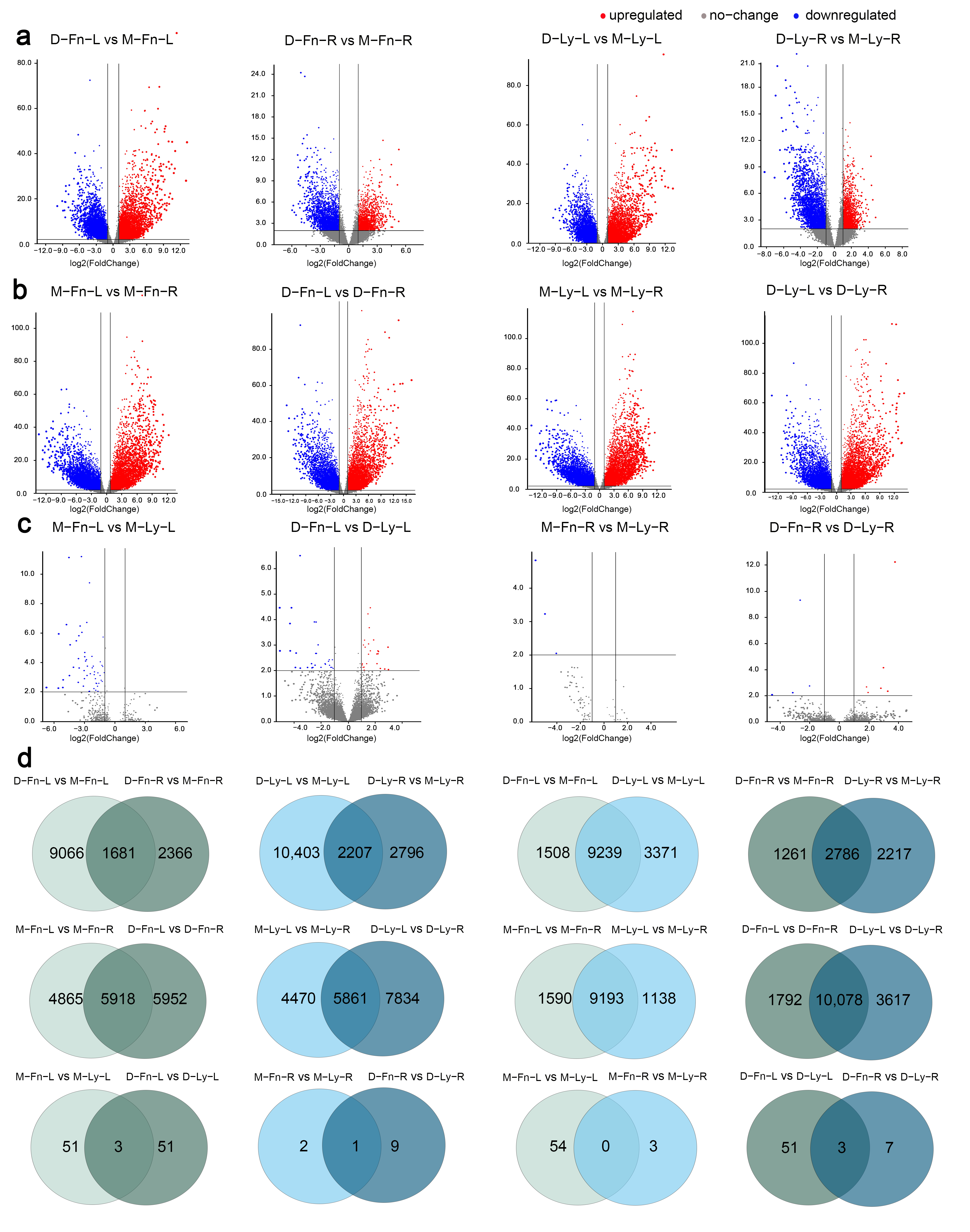

Supplement: Supplementary file 1 [file ijms-24-12019-s001.zip › Figure S1.tif]

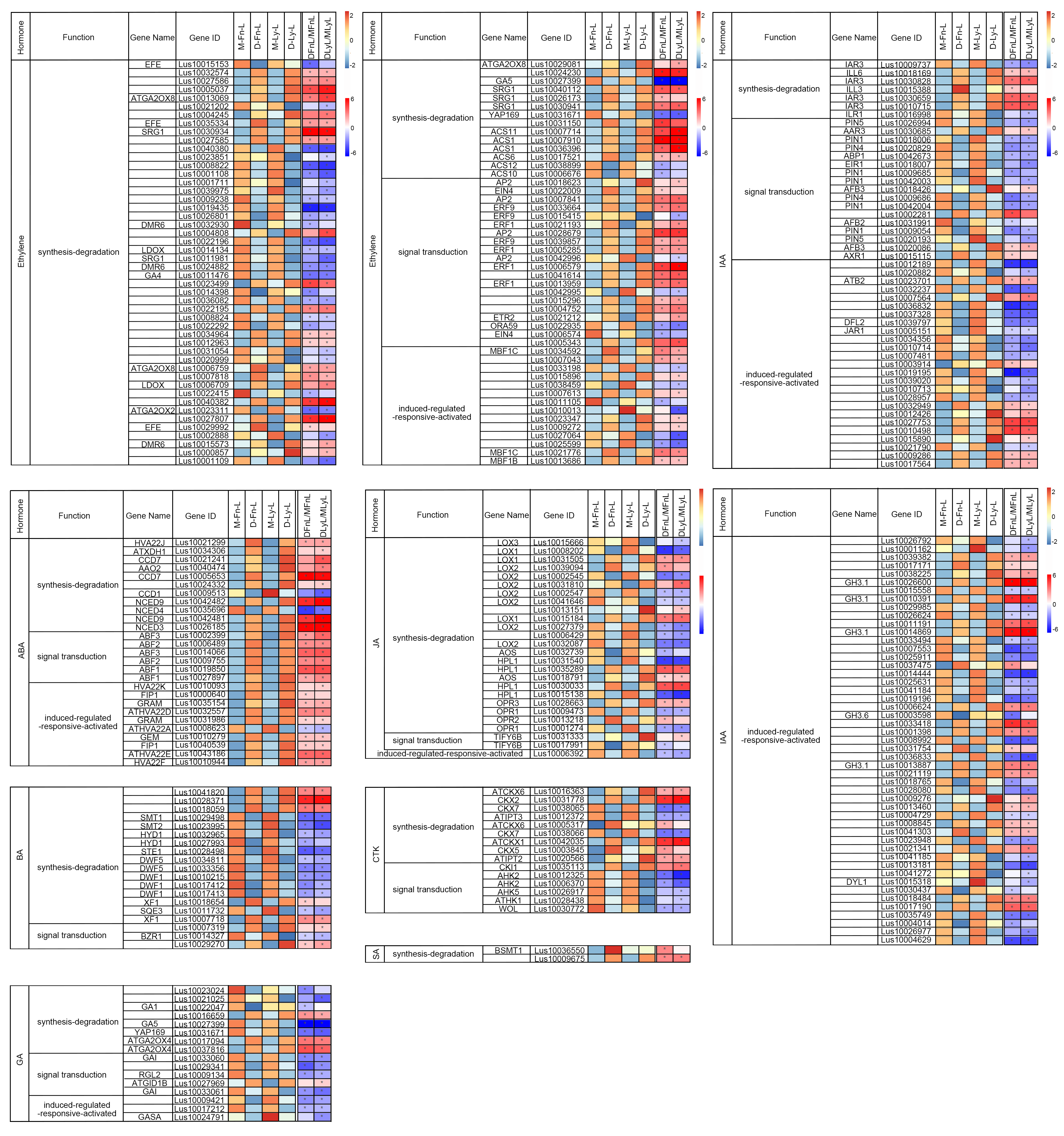

Supplement: Supplementary file 1 [file ijms-24-12019-s001.zip › Figure S2.tif]

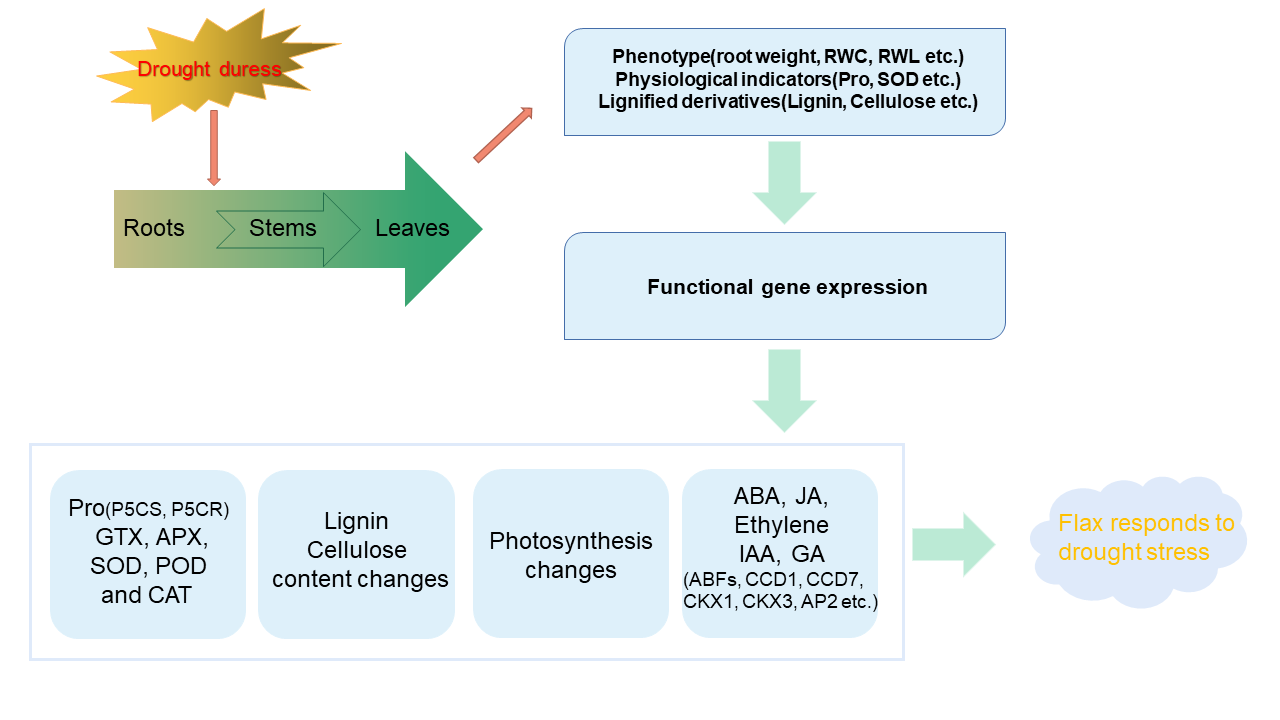

Supplement: Supplementary file 1 [file ijms-24-12019-s001.zip › Figure S3.TIF]
